# Stereoelectroencephalography electrode placement: Detection of blood vessel conflicts

**DOI:** 10.1111/epi.16294

**Published:** 2019-07-22

**Authors:** Kuo Li, Vejay N. Vakharia, Rachel Sparks, Roman Rodionov, Sjoerd B. Vos, Andrew W. McEvoy, Anna Miserocchi, Maode Wang, Sebastien Ourselin, John S. Duncan

**Affiliations:** ^1^ Department of Neurosurgery The First Affiliated Hospital of Xi'an Jiaotong University Xi'an China; ^2^ Department of Clinical and Experimental Epilepsy University College London London UK; ^3^ National Hospital for Neurology and Neurosurgery, Queen Square London UK; ^4^ Chalfont Centre for Epilepsy Chalfont UK; ^5^ School of Biomedical Engineering and Imaging Sciences St Thomas’ Hospital King's College London London UK; ^6^ Centre for Medical Image Computing University College London London UK

**Keywords:** computer‐assisted planning, segmentation, stereoelectroencephalography, vascular imaging

## Abstract

**Objective:**

Various forms of vascular imaging are performed to identify vessels that should be avoided during stereoelectroencephalography (SEEG) planning. Digital subtraction angiography (DSA) is the gold standard for intracranial vascular imaging. DSA is an invasive investigation, and a balance is necessary to identify all clinically relevant vessels and not to visualize irrelevant vessels that may unnecessarily restrict electrode placement. We sought to estimate the size of vessels that are clinically significant for SEEG planning.

**Methods:**

Thirty‐three consecutive patients who underwent 354 SEEG electrode implantations planned with computer‐assisted planning and DSA segmentation between 2016 and 2018 were identified from a prospectively maintained database. Intracranial positions of electrodes were segmented from postimplantation computed tomography scans. Each electrode was manually reviewed using “probe‐eye view” with the raw preoperative DSA images for vascular conflicts. The diameter of vessels and the location of conflicts were noted. Vessel conflicts identified on raw DSA images were cross‐referenced against other modalities to determine whether the conflict could have been detected.

**Results:**

One hundred sixty‐six vessel conflicts were identified between electrodes and DSA‐identified vessels, with 0‐3 conflicts per electrode and a median of four conflicts per patient. The median diameter of conflicting vessels was 1.3 mm (interquartile range [IQR] = 1.0‐1.5 mm). The median depth of conflict was 31.0 mm (IQR = 14.3‐45.0 mm) from the cortical surface. The addition of sulcal models to DSA, magnetic resonance venography (MRV), and T1 + gadolinium images, as an exclusion zone during computer‐assisted planning, would have prevented the majority of vessel conflicts. We were unable to determine whether vessels were displaced or transected by the electrodes.

**Significance:**

Vascular segmentation from DSA images was significantly more sensitive than T1 + gadolinium or MRV images. Electrode conflicts with vessels 1‐1.5 mm in size did not result in a radiologically detectable or clinically significant hemorrhage and could potentially be excluded from consideration during SEEG planning.

**Key Points**

- Various forms of vascular imaging are performed to identify vessels that should be avoided during SEEG planning
- We sought to estimate the size of vessels that are clinically significant for SEEG planning
- Conflicts with vessels 1‐1.5 mm in size were not associated with significant hemorrhage and could potentially be excluded from consideration during SEEG planning
- Addition of sulcal models as exclusion zones during planning would prevent the majority of electrode‐vessel conflicts across all vascular imaging modalities
- An important caveat is that in the absence of hemorrhages we were unable to identify the vessel diameter at which hemorrhage may result from electrode‐vessel conflicts

## 1 INTRODUCTION

Stereoelectroencephalography (SEEG) involves stereotactic placement of intracerebral electrodes to predefined targets to localize the epileptogenic zone and determine the possibility of resection to treat drug‐refractory focal epilepsy. Various forms of vascular imaging are performed to identify critical vasculature that should be avoided in SEEG.^1^ Digital subtraction angiography (DSA) is the gold standard for intracranial vascular imaging but is invasive and involves radiation exposure. Magnetic resonance (MR) imaging (MRI)‐based vascular imaging options including gadolinium‐enhanced T1 (T1 + Gad), MR venography (MRV), and MR angiography (MRA) have been used, without increased hemorrhage rates being reported.^2^

Once anatomical targets for SEEG sampling are identified, precise trajectory planning can be undertaken manually or using computer‐assisted planning (CAP).^3^, ^4^, ^5^ A safe trajectory should avoid critical vasculature and sulcal pial boundaries, avoid conflict with other electrodes, minimize the intracranial length, and maximize gray matter sampling while ensuring orthogonal drilling angles to the skull.

“Probe‐eye view” inspection of raw T1 + Gad MRI along the length of each trajectory may reveal vessels that were not identified by segmentation of the MRV/MRA. DSA identifies many more vessels than vascular MRI does. The raw DSA images are the most sensitive means of identifying blood vessels and therefore represent the gold standard.^6^, ^7^ It is unclear what the clinical significance is of the additional vasculature visualized by DSA. Showing small vessels that do not carry a risk of hemorrhage may make trajectory planning unnecessarily restrictive, but it is of course desirable to identify blood vessels that can cause clinically significant hemorrhage if damaged by an electrode. We therefore sought to estimate the size at which vessels become clinically significant for SEEG planning.

## 2 MATERIALS AND METHODS

Thirty‐three consecutive patients who underwent SEEG implantation were identified from a prospectively maintained database. All patients underwent SEEG implantation utilizing CAP with DSA‐based vessel segmentation between 2016 and 2018 at the National Hospital for Neurology and Neurosurgery, London, UK. A total of 354 electrodes were implanted (range = 7‐14 per patient). CAP was undertaken using the T1 + Gad as the reference image and segmented DSA models as critical structures to ensure a minimum distance of 3 mm between vasculature and calculate trajectories.

**Figure 1:**
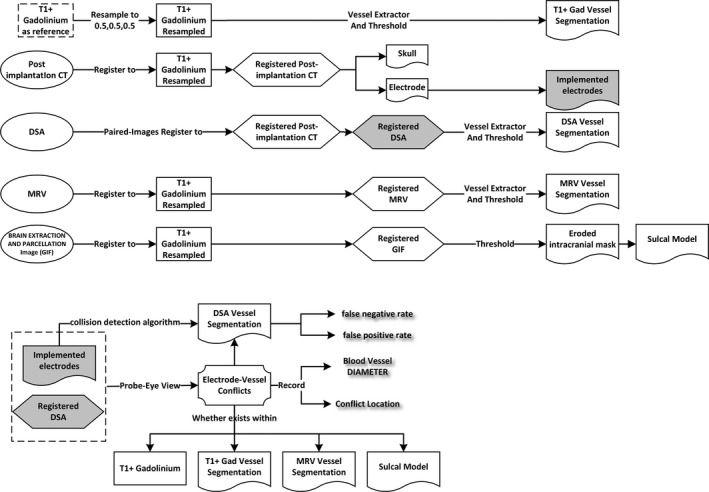
Schematic of image processing pipeline. T1 + gadolinium was used as a reference image (dashed rectangle), to which all raw imaging modalities (ovals) were registered (hexagons) to generate sulcal and vascular segmentation models (flags). Where conflicts (badge) between the implemented electrode and the digital subtraction angiography (DSA) vascular segmentation were identified, this was manually checked against the raw DSA acquisition, and false negative and false positive rates were calculated. Where conflicts were deemed to be true on the raw DSA, these were cross‐referenced with the other vascular segmentation models to determine whether these were also detectable. CT, computed tomography; GIF, geodesic information flow; MRV, magnetic resonance venography

The intracerebral vasculature was segmented from raw DSA (Siemens Somatom Definition AS, field of view [FOV] = 512 × 512 × 383, voxel size = 0.43 × 0.43 × 0.75 mm^3^), MRV/MRA (3T GE MR750, FOV = 220 × 220 × 148.8, voxel size = 0.43 × 0.43 × 0.60 mm^3^), and T1 + Gad (3T GE MR750, fast spoiled gradient‐echo, FOV = 256 × 256 × 220, voxel size = 1 × 1 × 1 mm^3^) images after application of an extraction filter.^8^ Geodesic information flow algorithms were used to generate a whole brain parcellation and pseudo–computed tomographic (CT) images (Centre for Medical Image Computing, University College London, UK),^9^ from which models for CAP were derived (Figure 1). The postimplantation CT was registered to the preoperative T1 + Gad reference image, and the electrode contacts were segmented to determine the implemented trajectory.

Implemented electrode segmentations from the postimplantation CT were coregistered and overlaid on the raw and segmented vascular images, and each trajectory was manually reviewed. An electrode‐vessel conflict was identified if there was overlap between the electrode segmentation and the vascular segmentation or if an unsegmented vessel could be manually identified from the raw vascular image. Due to the small amount of electrode bending, electrode contacts were linearly interpolated between contacts to identify electrode‐vessel conflicts within regions of the electrode that were radiotransparent on CT. All identified electrode‐vessel conflicts were manually verified on the raw DSA images.

All image processing and CAP were undertaken using the EpiNav (University College London, UK) platform. Each electrode was manually reviewed by scrolling along the trajectory using probe‐eye view with raw, unsegmented DSA images by two independent neurosurgeons. All conflicts between the electrodes and vasculature were recorded. The diameter of vessels was defined at the point of intersection between the electrode and the blood vessel. The location was defined as the distance along the trajectory from the cortical surface. The diameter of vessels, measured on the orthogonal projection of the raw DSA image, and the location were noted. Risk metrics for the implemented trajectories, calculated as the cumulative distance of the electrode from the vasculature on segmented DSA, were compared with manually identified locations of electrode‐vessel conflict, to determine the sensitivity of the automated collision detection algorithm.

Electrode‐vessel conflicts identified on raw DSA images were then reviewed on the corresponding raw and segmented T1 + Gad and MRV images to determine whether these could be identified. Sulcal models were extracted from whole brain geodesic information flow parcellations^10^ using the following automated method. First, a ray casting algorithm^11^ estimated the mean thickness of the cortical mantle. An intracerebral mask, eroded to below the level of the gyral crown, was then multiplied with the gray matter mask to create a sulcal model. The sulcal model was then used as an exclusion zone in addition to the T1 + Gad and MRV images to determine whether this could improve the sensitivity of the automated collision detection algorithm. Statistical analysis was carried out with SPSS Statistics 22.0 software (IBM). Electrode‐vessel conflicts between different vascular imaging modalities were compared by calculating the χ^2^ statistic with Bonferroni correction for multiple comparisons. This study was ethically approved by the Health Research Authority (12/LO/0377).

## 3 RESULTS

Thirty‐three patients (16 males) underwent a total of 354 electrodes planned with CAP and segmented DSA. In total, 166 vessel conflicts were identified on the raw DSA images. AdTech electrodes of varying length and contact number were implanted in all patients. Each electrode was found to have 0‐3 collisions with blood vessels. Each patient had a median of four electrode‐vessel conflicts (range = 1‐14). There were no clinically significant hemorrhages. The median diameter of conflicting vessels was 1.3 mm (interquartile range [IQR] = 1.0‐1.5 mm). The median depth of conflict was 31.0 mm (IQR = 14.3‐45.0 mm) from the cortical surface.

An automated collision detection algorithm employing the DSA vessel segmentation had a detection sensitivity of 72% (120/166) for electrode‐vessel conflict points, with a corresponding false negative rate of 28% (46/166) and false positive rate of 15% (21/141). In false negative cases, blood vessels that could be detected from visual inspection of the raw DSA were not segmented due to a low contrast to noise ratio, whereas in the false positive cases, the DSA vessel extraction filter artificially dilated the segmentation beyond the vessel dimensions on the raw DSA. As a result, the segmented DSA vasculature overlapped with the segmented electrode, but this was found not to be the case when cross‐referenced manually with the raw DSA imaging. When the MRV/MRA vessel segmentation was employed with the automated collision detection algorithm, it returned a detection sensitivity of 12% (20/166) for electrode‐vessel conflict points. From these 20 conflicts, MRV/MRA returned a false negative rate of 30% (6/20) and false positive rate of 0% (0/20). The T1 + Gad vessel segmentation had a detection sensitivity of 8% (14/166) for electrode‐vessel conflict points. From these 14 detected conflicts, there was an associated false negative rate of 50% (7/14) and false positive rate of 0% (0/14). Overall, 26.5% (44/166) of the electrode‐vessel conflicts were within the sulcal model. Using the sulcal model as an exclusion zone during CAP would therefore prevent these conflicts, but the clinical utility of this is unknown, as we have found that sulcal conflicts did not result in radiologically detectable hemorrhages. In addition, deep sulcal sampling is important for the detection of bottom of the sulcus focal cortical dysplasia.

Compared to the raw DSA, only 8% (14/166) and 4% (7/166) of the electrode‐vessel conflicts were detected using the MRV segmentation and T1 + Gad segmentations, respectively (χ^2^ = 69.9793, *P *<* *.0001). Combining a gray matter–derived sulcal exclusion model with the DSA, MRV, and T1 + Gad segmentations significantly increased the sensitivity of the automated collision detection algorithm from 72% to 83%, from 8% to 34%, and from 4% to 30%, respectively (*P *<* *.005) (Table 1).

**Table 1:** Detection of electrode‐vessel conflicts by vascular imaging method

	DSA		MRV		T1 + Gad	
Electrode‐vessel conflicts	Raw, reference	Segmentation	Raw	Segmentation	Raw	Segmentation
With sulcal model	100% (166/166)	83% (138/166)	40% (63/166)	34% (57/166)	34% (57/166)	30% (50/166)
Without sulcal model	100% (166/166)	72% (120/166)	12% (20/166)	8% (14/166)	8% (14/166)	4% (7/166)

Abbreviations: DSA, digital subtraction angiography; Gad, gadolinium; MRV, magnetic resonance venography.

## 4 DISCUSSION

A recent systematic review and meta‐analysis has shown that the prevalence of surgical morbidity following SEEG was one in 287 electrodes, equating to one in 29 patients. The most common complication associated with SEEG was intracranial hemorrhage, with an incidence of one in 316 electrodes.^12^ Due to underreporting of radiologically detected asymptomatic hemorrhages, the total hemorrhage rate is likely to be higher. The factors that determine the risk of bleeding are the distance of the planned trajectory from intracranial vasculature and the accuracy of implantation. Obtaining accurate vascular segmentation is an important factor in planning electrode trajectories through avascular corridors when both manual planning and CAP are undertaken. Here, we have shown that electrode‐vessel conflicts occurred frequently (166 times in 354 implanted SEEG electrodes) with small vessels (median diameter = 1.3 mm), and these did not result in any symptomatic hemorrhages These small vessel conflicts were detected from manual inspection of raw DSA images. Only 8% and 12% of these conflicts were detectable following manual inspection of the raw T1 + Gad and MRV/MRA images, respectively. We are unable to determine whether the electrodes displaced the vessels they conflicted with or whether they tamponaded them. Nevertheless, the results suggest that vessels of diameter = 1‐1.5 mm visualized on DSA could potentially be excluded from consideration during SEEG planning.

SEEG methodology was originally described by Talairach et al,^13^ in which the Talairach frame was used to insert electrodes using a double grid configuration. As this method was developed in the era before CT and MRI, planning was performed using coordinates derived from the Talairach atlas on the basis of the anterior commissure–posterior commissure line determined from ventriculography. Stereoscopic and stereotactic cerebral teleangiography was then used in combination to visualize the patient‐specific gyral/sulcal pattern and to define avascular trajectories.^14^ Due to the limitations imposed by the frame, the majority of the trajectories were in the axial plane. The implication is that to reach midline structures intracerebral sulci will have been transgressed in many of these cases. In the modern era, MRI has replaced ventriculography and the Talairach frame has been replaced by other stereotactic frames and/or robotic devices that have further improved the accuracy.^15^ Many institutions performing SEEG still undertake DSA for electrode planning, whereas others prefer gadolinium‐enhanced MR with or without additional noninvasive modalities such as MRV or catheter angiography.^1^

McGovern et al^16^ recently reported 549 SEEG implantations over an 8‐year period, with 19.1% implantations having hemorrhages seen on the postimplantation CT. DSA images have higher resolution than MRV and T1 + Gad and more sensitivity for detecting small blood vessels. Our results confirm that segmented DSA resulted in significantly greater vessel detection than segmentations derived from the T1 + Gad and MRV images.

CAP can only consider vessels that can be segmented, which represents a limited selection of the total number of vessels that can be visualized from the raw dataset. The role of CAP algorithms is to maximize the distance from intracerebral vasculature, among other parameters, along the entire length of the electrode. We have shown that the proportion of vessels conflicting with electrodes that can be segmented from T1 + Gad and MRV is 8% and 12%, compared to 72% with DSA. Consequently, CAP will result in a very high number of unintended vasculature conflicts if solely used with segmentations from T1 + Gad and MRV. Furthermore, given that the risk metric is calculated from segmented vessels, this would give a falsely reassuring risk score if DSA was not used.

**Figure 2:**
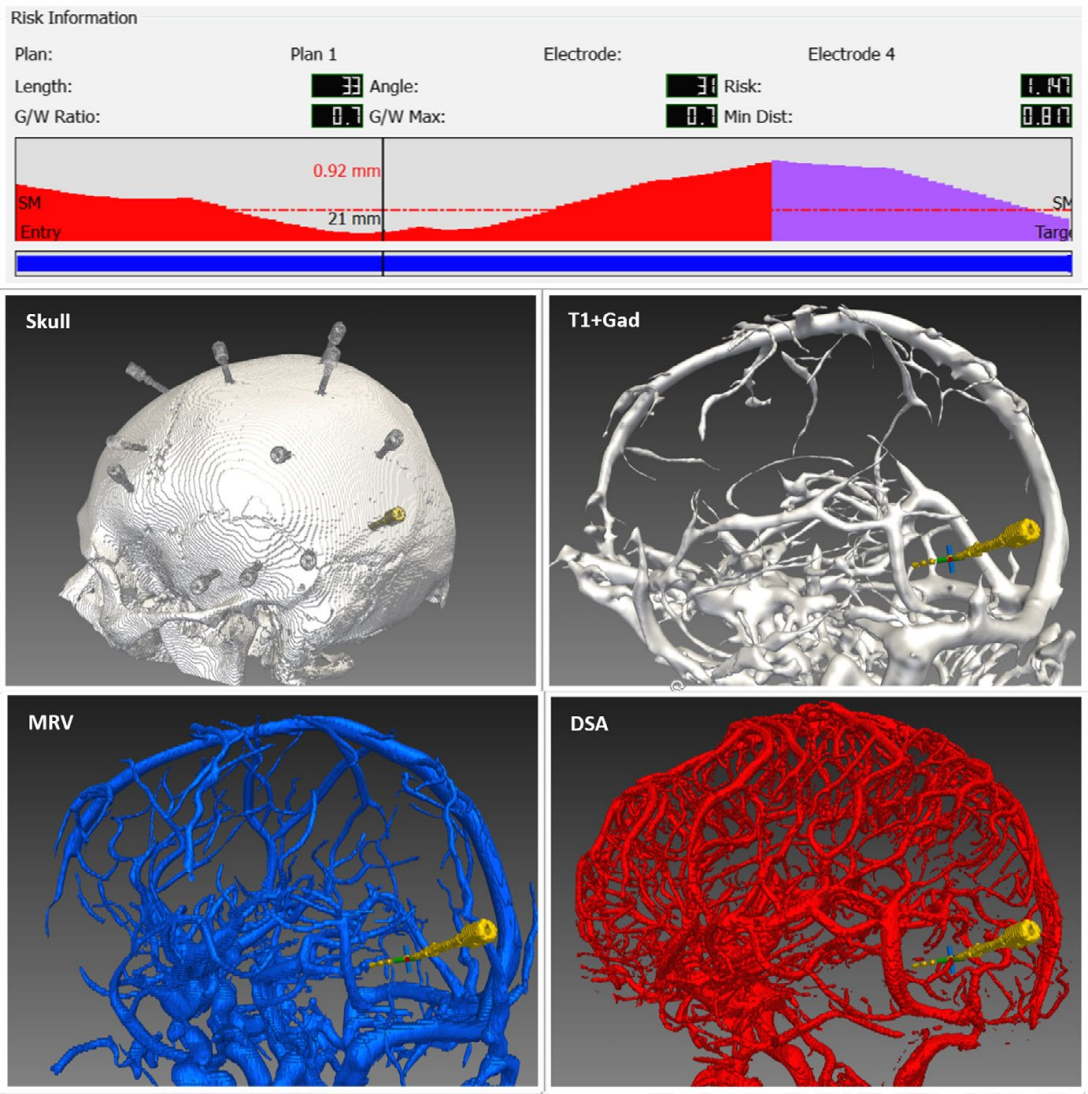
Example of a stereoelectroencephalographic electrode with T1 + gadolinium (Gad), magnetic resonance venography (MRV), and digital subtraction angiography (DSA) segmentation models. Risk metrics recorded for each electrode are provided as length (total intracerebral), angle (to skull), risk, gray/white (G/W) matter sampling ratio, and G/W maximum and minimum distance (Min Dist) from vasculature. For description of calculation of risk metrics, see Sparks et al.^19^ The schematic shows the automated collision detection algorithm depicting the distance from vasculature along the entire electrode. The safety margin (SM; dashed red line) was set at 3 mm. Where the distance of the electrode from vasculature falls below the SM, the distance from the vasculature is provided as the top value (0.92 mm) and the position along the electrode from the entry point as the bottom value (21 mm). Overall implantation is shown on the skull model. For clarity, only a single electrode segmentation is shown with the segmented T1 + Gad (white), segmented MRV (blue), and segmented DSA (red)

**Figure 3:**
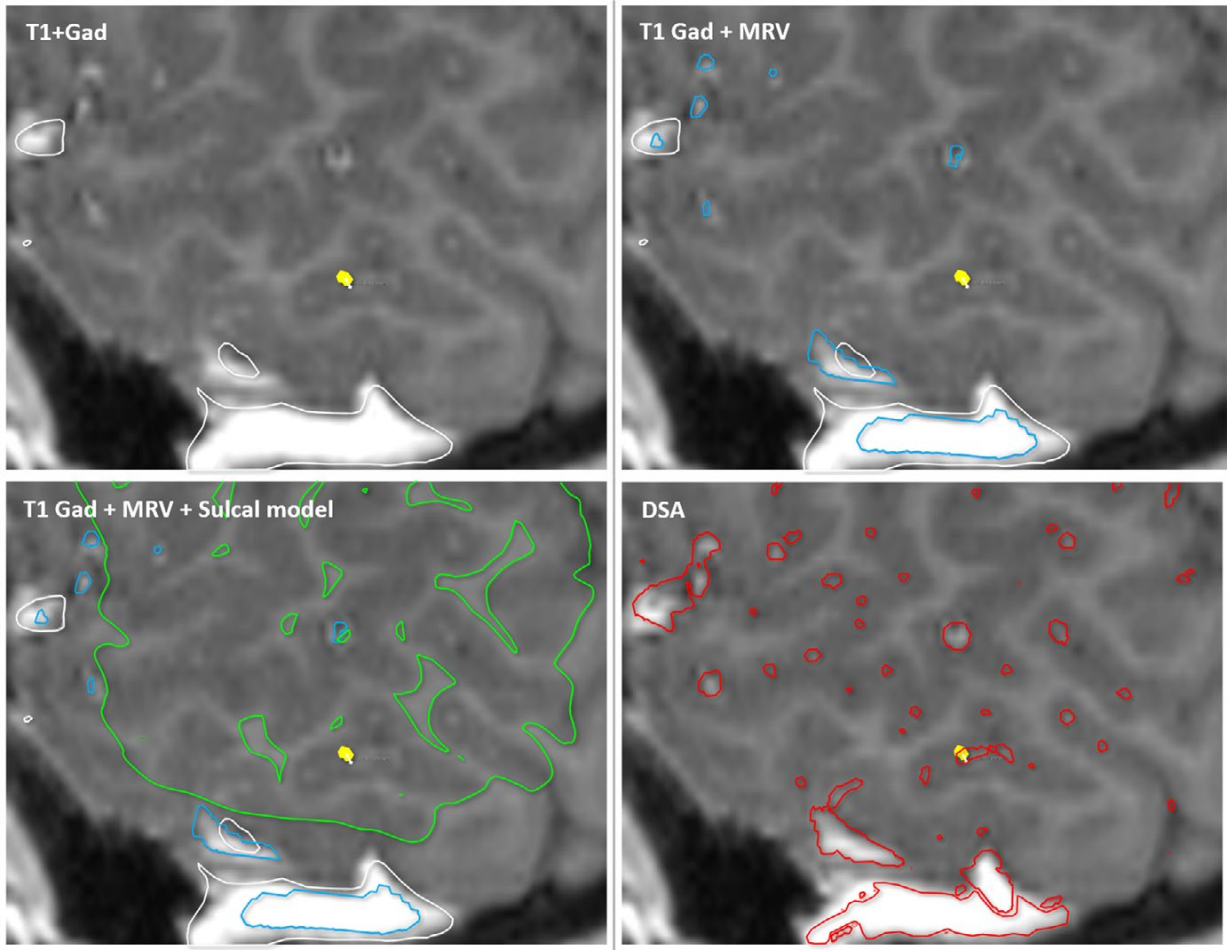
Effect of segmentation models on preventing vascular conflicts. The same electrode (gold) vessel conflict is depicted on T1 + gadolinium (Gad) with segmentations from T1 + Gad (white), magnetic resonance venography (MRV; blue), T1 + Gad with MRV and sulcal model (green), and digital subtraction angiography (DSA; red). The conflict was visible on the raw T1 + Gad, raw MRV (not shown), raw DSA (not shown), and DSA segmentation but could not be detected from the T1 + Gad and MRV segmentations. The use of a sulcal model as an exclusion zone with computer‐assisted planning would have prevented this conflict

Elias et al^17^ described a hemorrhagic complication rate of 10.1% in patients undergoing deep brain stimulation when an electrode transgressed a sulcus and 0.7% otherwise. We have therefore implemented a sulcal model and applied it as a critical structure. This improved the ability to detect electrode vessel conflicts from 72% to 83% with DSA vessel segmentation, from 8% to 34% with MRV vessel segmentation, and from 4% to 30% with T1 + Gad vessel segmentation. The implication is that the addition of a sulcal model may prevent vascular conflicts associated with CAP across all imaging modalities (Figures 2 and 3).

The median depth of conflict was 31.0 mm (IQR = 14.3‐45.0 mm) from the cortical surface, implying that most affected vessels were within sulci. In the CAP algorithm reported by De Momi et al,^5^ only vasculature within 25 mm of the surface was considered critical and the algorithm permitted conflicts with deeper vasculature. Other than anecdotal experience, we are not aware of any prospective validation showing that vasculature below a specified depth is less likely to bleed. The findings of our study may support this, however, as sulcal vessels are usually <1.3 mm in diameter, whereas cortical vessels tend to be larger.

**Figure 4:**
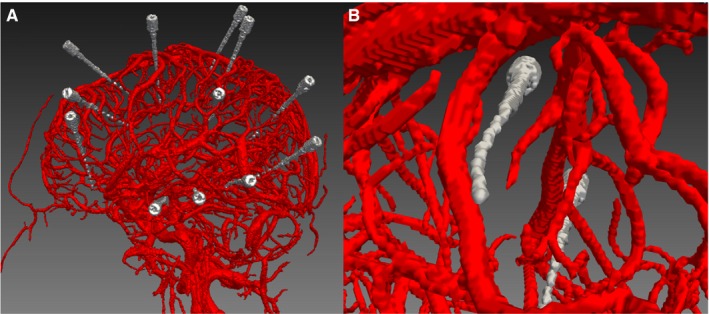
Inability to identify whether the vessels were venous or arterial in nature and whether vessels are displaced or transected by electrodes. A, The relationship between the preoperative digital subtraction angiography vessel segmentation and the postoperative electrodes. B, A closer view of the relationship between one electrode and a blood vessel at the point of conflict. It is difficult to determine the type of blood vessel, although in this case, the electrode seems to have been deflected by the vessel

The lack of any clinically significant intracerebral hemorrhage is an obvious limitation to the conclusions that can be drawn from this series, and we were therefore unable to assess the vessel size at which bleeding may occur. In view of the low incidence of symptomatic hemorrhage following SEEG of 2%‐3%, an observational study of this nature would require a prohibitively large sample size to draw significant conclusions. Animal studies would allow further inferences to be drawn. In addition, due to the nature of the acquisition of our DSA images, we were unable to determine whether the conflicting vessels were venous or arterial in nature. Due to the composition of the arterial wall, one would expect that these may be deflected by the electrode (Figure 4). On the other hand, due to the pressure of the blood in the arteries, an arterial bleed would be expected to have more significant sequelae. We are also unable to account for many other factors that affect the rate of bleeding, including the biomechanical properties of the stylet, difference in electrode rigidity between manufacturers, and whether these displace or pierce vessels.^18^

## 5 CONCLUSION

Following the implantation of 354 electrodes in 33 patients, we identified 166 electrode‐vessel conflicts based on the raw preoperative DSA. The median diameter of conflicting vessels was 1.3 mm, and none led to a clinically significant hemorrhage. It may therefore be possible to discount blood vessels with diameter up to 1‐1.5 mm from consideration during planning. These results may have implications for the choice of vascular imaging acquired during preoperative electrode planning if vessels down to 1.3 mm can be reliably visualized using noninvasive means. Furthermore, we show that the addition of a sulcal model results in a statistically significant increase in the detection of electrode‐vessel conflicts across all vascular imaging modalities. Due to the lack of clinically significant identified hemorrhages in this series, we were unable to identify the vessel diameter at which hemorrhage may result from electrode‐vessel conflicts.

## CONFLICT OF INTEREST

The authors have no personal, financial, or institutional interest in any of the drugs, materials, or devices described in this article. We confirm that we have read the Journal's position on issues involved in ethical publication and affirm that this report is consistent with those guidelines.
